# Genome-wide QTL mapping for stripe rust resistance in spring wheat line PI 660122 using the Wheat 15K SNP array

**DOI:** 10.3389/fpls.2023.1232897

**Published:** 2023-08-28

**Authors:** Qiong Yan, Guoyun Jia, Wenjing Tan, Ran Tian, Xiaochen Zheng, Junming Feng, Xiaoqin Luo, Binfan Si, Xin Li, Kebing Huang, Meinan Wang, Xianming Chen, Yong Ren, Suizhuang Yang, Xinli Zhou

**Affiliations:** ^1^ Wheat Research Institute, School of Life Sciences and Engineering, Southwest University of Science and Technology, Mianyang, Sichuan, China; ^2^ Department of Plant Pathology, Washington State University, Pullman, WA, United States; ^3^ Wheat Health, Genetics, and Quality Research Unit, US Department of Agriculture-Agricultural Research Service (USDA-ARS), Pullman, WA, United States; ^4^ Crop Characteristic Resources Creation and Utilization Key Laboratory of Sichuan Province, Mianyang Institute of Agricultural Science, Mianyang, Sichuan, China

**Keywords:** stripe rust, wheat, resistance, QTL mapping, yellow rust

## Abstract

**Introduction:**

Stripe rust is a global disease of wheat. Identification of new resistance genes is key to developing and growing resistant varieties for control of the disease. Wheat line PI 660122 has exhibited a high level of stripe rust resistance for over a decade. However, the genetics of stripe rust resistance in this line has not been studied. A set of 239 recombinant inbred lines (RILs) was developed from a cross between PI 660122 and an elite Chinese cultivar Zhengmai 9023.

**Methods:**

The RIL population was phenotyped for stripe rust response in three field environments and genotyped with the Wheat 15K single-nucleotide polymorphism (SNP) array.

**Results:**

A total of nine quantitative trait loci (QTLs) for stripe rust resistance were mapped to chromosomes 1B (one QTL), 2B (one QTL), 4B (two QTLs), 4D (two QTLs), 6A (one QTL), 6D (one QTL), and 7D (one QTL), of which seven QTLs were stable and designated as *QYrPI660122.swust-4BS*, *QYrPI660122.swust-4BL*, *QYrPI660122.swust-4DS*, *QYrPI660122.swust-4DL*, *QYrZM9023.swust-6AS*, *QYrZM9023.swust-6DS*, and *QYrPI660122.swust-7DS*. *QYrPI660122.swust-4DS* was a major all-stage resistance QTL explaining the highest percentage (10.67%–20.97%) of the total phenotypic variation and was mapped to a 12.15-cM interval flanked by SNP markers *AX-110046962* and *AX-111093894* on chromosome 4DS.

**Discussion:**

The QTL and their linked SNP markers in this study can be used in wheat breeding to improve resistance to stripe rust. In addition, 26 lines were selected based on stripe rust resistance and agronomic traits in the field for further selection and release of new cultivars.

## Introduction

Wheat stripe rust, caused by *Puccinia striiformis* Westend. f. sp. *tritici* Erikss. (*Pst*), is one of the most destructive diseases in the world (Chen, 2005; Milus et al., 2009). Losses from stripe rust typically range from 10% to 70% in commercial production environments, depending on the cultivar, prevailing climatic conditions, and inoculum pressure (Bariana et al., 2016; Zhou et al., 2022a). However, the disease can cause a 100% loss of yield for susceptible varieties (Bariana et al., 2016). Since 1950, the disease has occurred on an annual average of 4 million hectares in China. In particular, the five major outbreaks of wheat stripe rust in 1950, 1964, 1990, 2002, and 2017 all occurred on over 5.5 million hectares, resulting in a loss of 13.8 million tons of yield (Li and Zeng, 2000; Wan et al., 2004; Huang et al., 2018). Stripe rust can be controlled by resistant cultivars, fungicides, and some cultural practices. Compared to other approaches, planting resistant cultivars has been proven to be the most effective, easy-to-use, economical, and environmentally friendly way to control disease (Line, 2002; Chen, 2005).

Depending on phenotypic performance at different growth stages, wheat rust resistance can be classified into two types: all-stage resistance (ASR) and adult plant resistance (APR), sometimes also known as high-temperature adult plant (HTAP) resistance (Qayoum and Line, 1985; Chen, 2005; Lin and Chen, 2007; Rosewarne et al., 2013). All-stage resistance, also known as seedling resistance, can be detected at the seedling stage but is expressed at all growth stages. Such resistance is often race-specific and, thus, easily overcome by virulent races (Line, 2002; Chen, 2005; Chen, 2013). Due to race specificity, ASR often fails within 3–5 years of deployment (Jambuthenne et al., 2022). In contrast, HTAP resistance becomes more effective as plants grow older and the weather becomes warmer. It is usually non-race-specific, quantitatively inherited, and more likely to be durable. However, HTAP resistance is mostly incomplete, and the level is influenced by plant growth stage, temperature, and disease pressure (Chen, 2013).

To date, 84 permanently named and a large number of temporarily designated stripe rust resistance genes (*Yr* genes) and quantitative trait loci (QTLs) have been reported in wheat (Klymiuk et al., 2022). These resistance genes come mainly from common wheat cultivars, local germplasm, and wild relatives. Among them, *Yr5*/*Yr7*/*YrSP*, *Yr10*, *Yr15*, *Yr18*, *Yr36*, *Yr46*, *YrU1*, and *YrAS2388* were cloned and characterized (Fu et al., 2009; Krattinger et al., 2009; Liu et al., 2014; Moore et al., 2015; Klymiuk et al., 2018; Marchal et al., 2018; Zhang et al., 2019; Wang et al., 2020). Most ASR genes have been overcome by virulent races. ASR genes *Yr5* and *Yr15* are still effective against all *Pst* races identified in the United States and many other countries (Wang and Chen, 2017). However, races virulent on *Yr5* gene have been reported in Australia, India, China, and Turkey (Wellings and McIntosh, 1990; Zhang et al., 2020; Tekin et al., 2021), and the *Yr15* virulence has been documented in Afghanistan (Gerechter-Amitai et al., 1989). Most wheat cultivars that have shown durable resistance to stripe rust have APR or HTAP resistance controlled by variable numbers of genes or QTL (Lin and Chen, 2007; Lin and Chen, 2008b; Santra et al., 2008; Carter et al., 2009; Paillard et al., 2012; Ren et al., 2012a; Lu et al., 2014; Zhou et al., 2014; Dong et al., 2017; Feng et al., 2018; Liu et al., 2018; Liu et al., 2019; Liu et al., 2020). In order to obtain a high degree of durable resistance, combining the two types of resistance types in the same background is considered a preferred method to improve the resistance to stripe rust in wheat breeding (Chen, 2007; Ren et al., 2012a; Chen, 2013; Liu et al., 2018).

The development of molecular markers, especially single-nucleotide polymorphism (SNP) markers, has revolutionized QTL analysis. A SNP is caused by a single-nucleotide mutation due to the insertion, deletion, and replacement of a single base segment in the genome. SNPs exist in the entire genomes of biological individuals and are the most abundant. SNP markers are now widely used in genetic analysis and breeding (Ma et al., 2019). Recent advances in sequencing technology have led to the availability of many SNP arrays in wheat (Rasheed et al., 2017). High-throughput genotyping techniques, including Wheat 9K (Cavanagh et al., 2013), 15K (Soleimani et al., 2020), 90K (Wang et al., 2014; Wu et al., 2018), 660K (Cui et al., 2017), and 820K SNP (Winfield et al., 2016) arrays, are now available. Among these SNP arrays, the 15K array is generally adequate and cost-effective for mapping traits of interest (Soleimani et al., 2020).

PI 660122, a spring wheat germplasm, was developed by the Wheat Health, Genetics, and Quality Research Unit of the US Department of Agriculture, Agricultural Research Service (USDA-ARS), and Washington State University and deposited in the USDA-ARS National Small Grains Collections (NSGC). In previous studies, the germplasm showed a high level of resistance in field tests over multiple years (Wang et al., 2012; Zhou et al., 2015b). At the seedling stage, it was resistant to US races PST-43 and PST-127 and Chinese races CYR29, CYR31, CYR32, and CYR33 and moderately resistant to US races PST-100 and PST-114 and Chinese race PST-HY8 of *Pst* (Wang et al., 2012; Zhou et al., 2015b). A comparison of greenhouse and field tests indicated that PI 660122 had effective ASR and possible HTAP resistance. The objectives of the present study were to further characterize the stripe rust resistance in PI 660122, map QTL for ASR and APR, and identify the QTL by comparing their chromosomal locations with previously reported stripe rust resistance QTL.

## Materials and methods

### Plant materials

To map the QTL for stripe rust resistance in PI 660122, we developed a mapping population from a cross between Zhengmai (ZM9023, as the female parent) and PI 660122 (as the male parent). PI 660122 was developed from cross Avocet S/PI 610755 (Wang et al., 2012). Avocet S (AvS), an Australian spring wheat selection, is highly susceptible to most *Pst* races in China and many other countries and has been used as a susceptible control in stripe rust tests. PI 610755 is a Mexico spring wheat variety, selected from the cross Altar 84/*Aegilops tauschii (191)*//Opata M85. ZM9023, a spring wheat cultivar developed by the Wheat Research Institute of Henan Academy of Agricultural Sciences, is moderately or highly susceptible to the currently predominant *Pst* races in China (Xue et al., 2014). We developed a total of 239 F_5_ and F_6_ recombinant inbred lines (RILs) from the ZM9023 × PI 660122 cross, using the single-seed descent method.

### Greenhouse tests

Seedling tests were conducted in a greenhouse to evaluate the stripe rust responses of PI 660122 and Zhengmai 9023. For each genotype, 10–12 seeds were seeded in a 9 cm × 9 cm × 9 cm plot. At the one-leaf stage, seedlings were uniformly inoculated with fresh urediniospores of a *Pst* race mixed with talc at a ratio of 1:50. Three Chinese *Pst* races, CYR31, CYR32, and CYR34, were used in the seedling tests. Inoculated seedlings were kept in a dew chamber in the dark at 8°C and above 100% relative humidity for 24 h. The seedlings were then moved to a growth chamber at 16°C with a daily 16-h light for stripe rust development. The infection type (IT) data were recorded 18 days to 21 days after inoculation using the 0–9 scale (Line and Qayoum, 1992). Seedlings of AvS were included as the susceptibility check in each race test. Later, 15 RILs selected for each containing only one QTL were also tested together with the parents with the three races at the seedling stage in the greenhouse under the same conditions.

### Field tests

The F_5_ and F_6_ RILs and their parents were tested for stripe rust responses to stripe rust in the experimental fields in Mianyang (MY; 31°33′N, 104°55′E) in 2021 (21) and both MY and Guangyuan (GY; 22:32°14′N, 106°17′E) in the Sichuan Province in 2022 (22). The field tests were conducted with one replicate at 21MY and 22GY and two replicates (completely randomized block design) at 22MY based on the available seed quantity. Each plot consisted of a single row, 1.0 m in length and with 25 cm between rows. Approximately 20 to 30 seeds were sown in each row. AvS was planted in a row every 20 rows as a susceptible check and spore spreader for increasing stripe rust pressure and uniformity in the nursery. To increase the *Pst* inoculum, AvS was also planted around the nursery. MY and GY are ideal regions for stripe rust, as *Pst* can over-winter and over-summer, and the nursery was naturally infected without artificial inoculation (Zhou et al., 2019).

The stripe rust IT of each parent or RIL was rated on a scale of 0–9 (Line and Qayoum, 1992). Disease severity (DS) was scored using a modified scale as previously described (Lin and Chen, 2007). Both IT and DS data were collected twice in each season. The first record was taken when susceptible AvS showed approximately 80% severity, and the second was approximately a week later (Nsabiyera et al., 2018). Agronomic traits such as plant height (PH), spike length (SL), productive tiller number (PTN), kernels per spike (KPS), and thousand-grain weight (TGW) were determined to select RILs. PH was measured from the ground to the top of the spike excluding awn after the milking stage; KPS, SL, and PTN of each plant were counted at maturity; TGW was measured after harvest.

### DNA extraction and genotyping

Fresh young leaves of PI 660122, ZM9023, and 239 F_5_ RILs were harvested from the experimental field in January 2021. DNA from the fresh leaves was extracted using a modified cetyltrimethyl ammonium bromide (CTAB) method (Li et al., 2013). DNA was dissolved in ddH_2_O (100 μL), and DNA quality and concentration were determined by spectrophotometry (NanoDrop ND-1000, Thermo Scientific, Wilmington, DE, USA) after the DNA. DNA stock solutions were diluted with sterilized ddH_2_O to different concentrations according to individual experimental requirements for molecular analyses.

The parents and the 239 RILs were genotyped by China Golden Marker (Beijing) Biotech Co., Ltd. (http://www.cgmb.com.cn/) using the 15K SNP chip (Soleimani et al., 2020).

### Statistical analysis, genetic map construction, and QTL mapping

Analysis of variance (ANOVA) and analysis of Pearson’s correlation coefficients were performed to analyze the stripe rust phenotypic data using the “AOV” tool in the QTL Ici Mapping V4.2 software (Wang, 2009; Meng et al., 2015). The same software was also used to analyze the genotypic data. After the genotypic data were scanned for missing and undetected data, redundant markers were deleted using the “Bin” function. Genetic maps were constructed using the Kosambi mapping function (Kosambi, 2016). QTL mapping was performed using the genetic maps and the IT and DS data based on inclusive composite interval mapping (ICIM) with preset parameters Step = 1 cM, value *p* for input variables (PIN) = 0.0001, and logarithm of odds (LOD) = 2.5. A QTL was identified when the logarithm of odds (LOD) score was greater than 2.5. To determine the additive effects of QTL, the effects of QTL combinations were demonstrated by plotting box plots for mean IT and mean DS of RILs sharing the same number of beneficial alleles.

## Results

### Stripe rust responses of the parents and RILs

**Figure 1:**
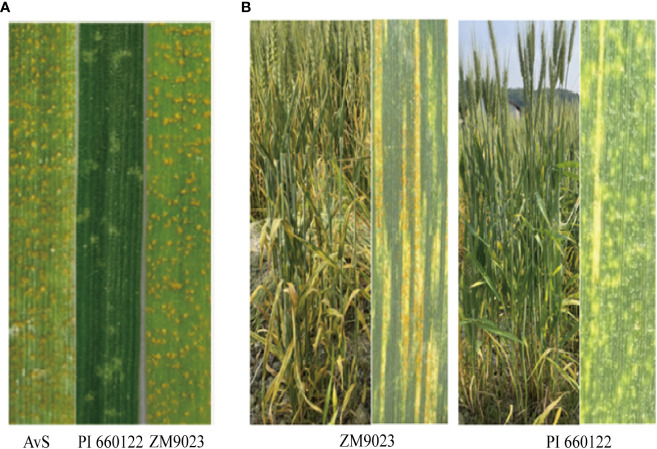
Stripe rust response of resistant parent PI 660122, susceptible parent Zhengmai 9023 (ZM9023), and susceptible check AvS with Chinese race CYR34 of *Puccinia striiformis* f sp. *tritici* at the seedling stage **(A)** and stripe rust reactions on flag leaves of ZM9023 and PI 660122 **(B)**.

In the greenhouse seedling tests, PI 660122 was highly resistant (IT of 2) to the tested three Chinese *Pst* races, whereas Zhengmai 9023 was highly susceptible (IT of 8–9) similar to the susceptible check AvS (**Figure 1A**). In the field tests under natural *Pst* infection, the final adult plant IT of PI 660122 was 2 across the two years and two locations, and its DS ranged from 5% to 10%, (**Figure 1B**). In contrast, Zhengmai 9023 was moderately resistant (IT of 5–6) with DS of 40%–50%. For comparison, AvS had IT of 9 and DS of 100%.

**Figure 2:**
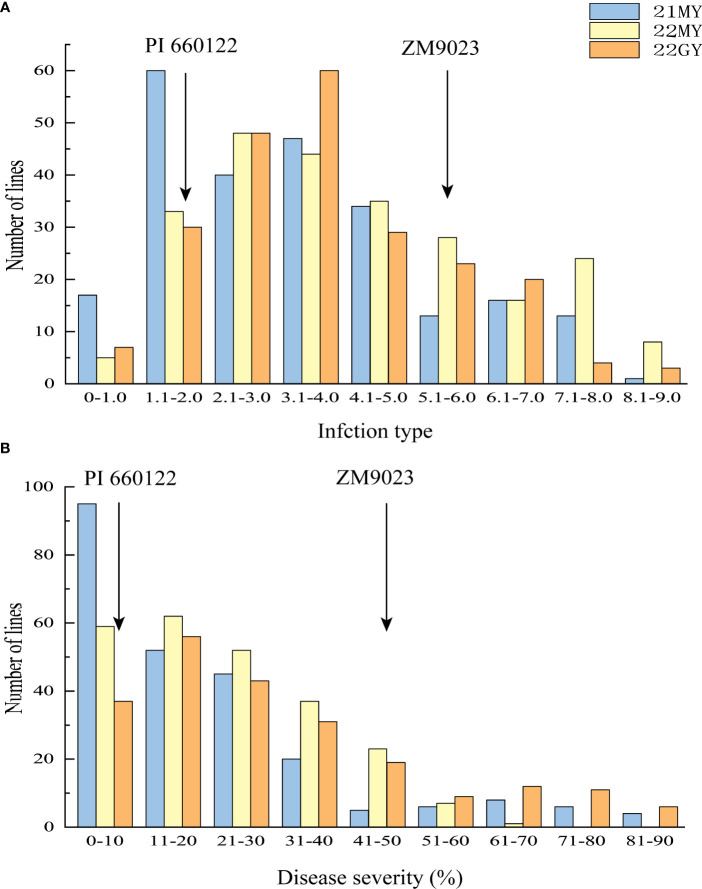
Frequency distributions of mean infection types (IT) **(A)** and disease severity (DS) **(B)** for 239 F_6_ RILs from cross Zhengmai 9023 × PI 660122 tested in Mianyang (MY) in 2021 (21) and 2022 (22) and Guangyuan (GY) in 2022. Arrows indicate the values of the parent lines. RILs, recombinant inbred lines.

The RIL population had ITs ranging from 0 to 9 and DS from 0 to 90% across the years and locations (**Figure 2**). The IT and DS data from both sites and from both 2021 and 2022 at MY were each highly correlated (r = 0.76–0.81, *p* < 0.001 for IT; r = 0.61–0.75, *p* < 0.001 for DS) (**Table 1**). The ANOVA results showed significant variations (*p* < 0.001) among RILs, environments, and line × environment interactions for both IT and DS. The stripe rust phenotypes were influenced more by the environment than by the interaction of line and environment. The broad-sense heritability (*h* ^2^) was estimated at 0.92 using the IT data and 0.86 based on the DS data across the two sites (**Table 2**).

**Table 1:** Correlation coefficients (r) of infection type (IT) and disease severity (DS) of the recombinant inbred lines Zhengmai 9023 × PI 660122 tested in different environments.

Environment^a^	21MY	22MY	22GY
21MY	NA^b^		
22MY	0.81 (0.69)***^c^	NA	
22GY	0.77 (0.61)***	0.76 (0.76)***	NA

a 21, 2021; 22, 2022; MY, Mianyang; GY, Guangyuan.

b NA, not applicable.

c The r values based on DS data are given in parentheses.

“***” denotes the r value is significant at p < 0.001.

**Table 2:** Analysis of variance and estimate of broad-sense heritability (*h* ^2^) of infection type (IT) and disease severity (DS) in the recombinant inbred line (RIL) population of Zhengmai 9023 × PI 660122 tested at Mianyang in 2021 and 2022 and Guangyuan in 2022.

Source of variation	DS			IT		
	*df* ^a^	Mean square	F value	*df*	Mean square	F value
Lines	238	1,427.69	44.51***^b^	238	18.17	56.86***
Environments	2	7,683.04	239.53***	2	64.90	203.16***
Line × Environment	459	224.64	7.00***	459	1.65	5.17***
Error	689	32.08		689	0.32	
*h* ^2^	0.86			0.92		

a df, degree of freedom.

b “***” denotes the significance level of p < 0.001.

### Genetic linkage map construction

A total of 5,432 SNPs in the 15K SNP array showed homozygous polymorphisms between the two parents. After the redundant markers were filtered out, 4,102 SNPs with known chromosome locations were obtained and used as inputs in the linkage analysis using QTL Ici Mapping V4.2. The 4,102 SNPs covered a total map length of 7,937.6 cM, with the genetic length ranging from 135.6 cM for chromosome 1B to 635.5 cM for chromosome 5A (**Table 3**). The number of markers per chromosome ranged from 60 for chromosome 6A to 322 for chromosome 2A, with an average of 189 SNPs. The mean distance between adjacent SNP markers ranged from 0.5 cM for chromosome 1B to 7.3 cM for chromosome 2D, with an overall mean of 1.9 cM. Genomes A, B, and D included 1,374 (33.50%), 1,672 (40.76%), and 1,056 (25.74%) SNPs covering lengths of 2,630.1 cM, 2,144.1 cM, and 3,163.4 cM with mean marker distance of 1.91 cM, 1.28 cM, and 3.00 cM, respectively. The map was used to identify significant associations between SNPs and stripe rust resistance.

**Table 3:** Summary of chromosome assignment, number of SNPs, map length, and marker density of the genetic maps of the Zhengmai 9023 × PI 660122 recombinant inbred population.

Chromosome	No. of SNPs	Map length (cM)	Mean SNP distance (cM)
1A	156	311.4	2.0
1B	256	135.6	0.5
1D	82	561.9	6.9
2A	322	379.2	1.2
2B	254	339.4	1.3
2D	197	465.3	7.3
3A	249	362.0	1.5
3B	194	318.6	1.6
3D	190	428.1	2.3
4A	179	296.5	1.7
4B	193	281.5	1.5
4D	71	235.9	3.3
5A	235	635.5	2.7
5B	223	368.9	1.7
5D	138	500.5	3.6
6A	60	288.0	4.8
6B	303	390.1	1.3
6D	109	440.4	4.0
7A	173	357.5	2.1
7B	249	310.0	1.2
7D	269	531.3	2.0
Total	4102	7937.6	1.9
Average	195	376.7	1.9

SNPs, single-nucleotide polymorphisms.

### QTL analysis of stripe rust resistance

QTL scans on all 21 chromosomes were performed using the ICIM method in the software QTL Ici Mapping V4.2. A total of nine QTLs contributing to stripe rust resistance in the Zhengmai 9023 × PI 660122 RIL population were identified with one QTL each on chromosomes 1B, 2B, 6A, 6D, and 7D and two QTLs each on 4B and 4D. Then, ICIM, single-marker analysis (SMA), and ICIM epistatic QTL (ICIM-EPI) for epistatic mapping were performed on QTL chromosome regions of these chromosomes. Among the QTL, seven (*QYrPI660122.swust-4BS*, *QYrPI660122.swust-4BL*, *QYrPI660122.swust-4DS*, *QYrPI660122.swust-4DL*, *QYrZM9023.swust-6AS*, *QYrZM9023.swust-6DS*, and *QYrPI660122.swust-7DS*) were detected in all environments, and two (*QYrZM9023.swust-1BL* and *QYrPI660122.swust-2BL*) were only detected in 22MY. Of the nine QTLs, six (*QYrPI660122.swust-2BL*, *QYrPI660122.swust-4BS*, *QYrPI660122.swust-4BL*, *QYrPI660122.swust-4DS*, *QYrPI660122.swust-4DL*, and *QYrPI660122.swust-7DS*) were from PI 660122 and three (*QYrZM9023.swust-1BL*, *QYrZM9023.swust-6AS*, and *QYrZM9023.swust-6DS*) from Zhengmai 9023.

**Figure 3:**
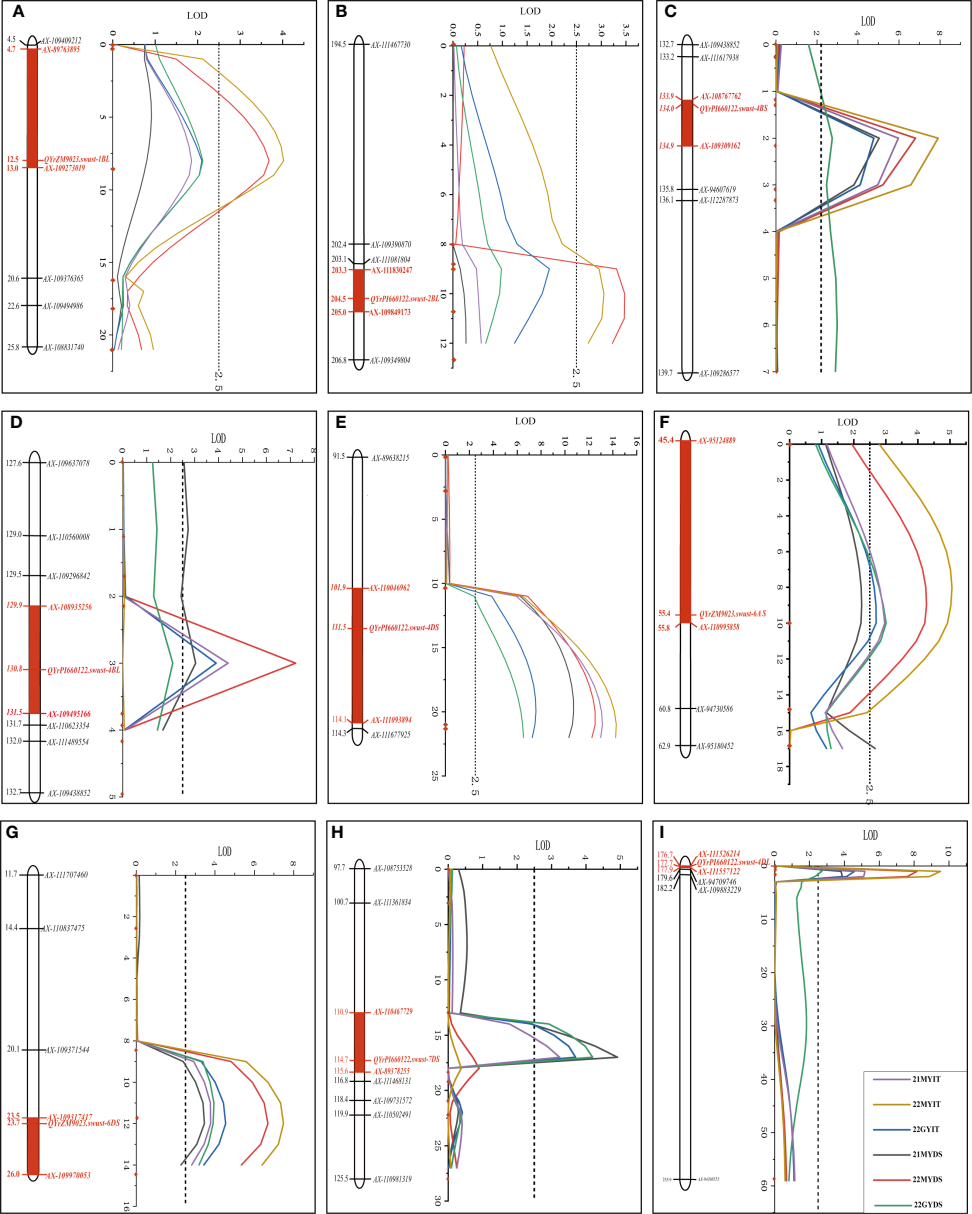
Stripe rust resistance QTLs on the genetic map of chromosomes 1BL **(A)**, 2BL **(B)**, 4BS **(C)**, 4BL **(D)**, 4DS **(E)**, 6AS **(F)**, 6DS **(G)**, 7DS **(H)**, and 4DS **(I)** based on infection type (IT) and disease severity (DS) data. The y-axis is in centimorgan (cM) distance, and the x-axis denotes LOD value. The red rectangle on the genetic map indicates the corresponding QTL region. QTLs, quantitative trait loci; LOD, limit of detection.

*QYrZM9023.swust-1BL*, located at an 8.27-cM interval spanned by SNP markers *AX-89763895* and *AX-109273019*, explained 7.41% and 7.29% of phenotypic variation in IT and DS, respectively, and was detected only in 22MY. *QYrPI660122.swust-2BL*, located at a 1.83-cM interval spanned by SNP markers *AX-109849173* and *AX-109349804*, explained 4.65% and 5.57% of phenotypic variation in IT and DS, respectively, and was only detected in 22MY. *QYrPI660122.swust-4BS*, located at a 0.96-cM interval spanned by SNP markers *AX-108767762* and *AX-109309162*, explained 10.94%–15.00% and 5.84%–12.93% of phenotypic variation in IT and DS, respectively, across all environments. *QYrPI660122.swust-4BL*, mapped to a 1.54-cM interval flanked by SNP markers *AX-108935256* and *AX-108984536*, explained 8.25%–9.09% and 5.75%–13.51% phenotypic variation in IT and DS, respectively, and was detected in all environments. *QYrPI660122.swust-4DS*, located at a 12.15-cM interval spanned by SNP markers *AX-110046962* and *AX-111093894*, explained 11.64%–17.20% and 13.22%–20.97% phenotypic variation in IT and DS, respectively, across all environments. *QYrPI660122.swust-4DL*, located at a 1.23-cM interval spanned by SNP markers *AX-94560848* and *AX-111557122*, explained 11.18%–18.24% and 6.60%–17.37% phenotypic variation in IT and DS, respectively, across all environments. *QYrZM9023.swust-6AS*, located at a 10.37-cM interval spanned by SNP markers AX-95124889 and *AX-110995858*, explained 5.43%–9.11% and 6.23%–7.92% of phenotypic variation in IT and DS, respectively, across all environments. *QYrZM9023.swust-6DS*, located at a 2.58-cM interval spanned by SNP markers *AX-11475193* and *AX-109317417*, explained 7.24%–13.33% and 7.25%–12.22% of phenotypic variation in IT and DS, respectively, across all environments. *QYrPI660122.swust-7DS*, located at a 4.75-cM interval spanned by SNP markers *AX-110467729* and *AX-89378255*, explained 11.64%–17.20% and 13.22%–20.97% of phenotypic variation in IT and DS, respectively, and was detected in two environments, 21MY and 22GY (**Figure 3**, **Table 4**).

**Table 4:** Summary of nine stripe rust resistance QTLs identified based on mean disease severity (DS) and infection type (IT) of 239 RILs from Zhengmai 9023 × PI 660122 cross-tested in Mianyang 2021–2022 and Guangyuan 2022.

QTL	Environment	Center marker	Right marker	IT			DS		
				LOD^a^	PVE^b^	Add^c^	LOD	PVE	Add
*QYrZM9023.swust-1BL*	22MY	*AX-89763895*	*AX-109273019*	4.02	7.41	0.57	3.80	7.29	3.75
*QYrPI660122.swust-2BL*	22MY	*AX-109849173*	*AX-109349804*	2.52	4.65	−0.45	3.00	5.57	−.3.29
*QYrPI660122.swust-4BS*	21MY	*AX-108767762*	*AX-109309162*	5.97	14.15	−0.64	5.03	10.36	−5.69
	22MY			7.91	15.00	−0.76	6.72	12.76	−4.82
	22GY			4.77	10.940	−0.53	2.75	5.51	−4.84
*QYrPI660122.swust-4BL*	21MY	*AX-109495166*	*AX-108935256*	4.40	8.25	−0.56	3.04	5.75	−4.50
	22MY			–	–	–	7.19	13.51	−4.99
	22GY			3.90	9.09	−0.48	–	–	–
*QYrPI660122.swust-4DS*	21MY	*AX-110046962*	*AX-111093894*	13.13	21.82	−0.99	10.72	18.11	−8.83
	22MY			14.29	23.78	−1.06	12.65	21.04	−7.03
	22GY			7.57	13.81	−0.74	6.53	12.58	−7.57
*QYrPI660122.swust-4DL*	21MY	*AX-111526214*	*AX-111557122*	5.18	11.18	−0.60	3.81	7.22	−5.05
	22MY			9.51	18.23	−0.84	8.20	17.36	−5.33
	22GY			4.58	12.71	−0.52	2.71	6.60	−4.83
*QYrZM9023.swust-6AS*	21MY	*AX-95124889*	*AX-110995858*	2.97	5.53	0.46	–	–	–
	22MY			5.06	9.11	0.67	4.39	7.92	4.17
	22GY			2.70	5.43	0.41	3.01	6.23	5.13
*QYrZM9023.swust-6DS*	21MY	*AX-109317417*	*AX-111475193*	3.78	7.24	0.52	3.47	7.26	4.80
	22MY			7.48	13.33	0.75	6.85	12.44	4.87
	22GY			4.54	9.39	0.52	3.91	7.79	5.76
*QYrPI660122.swust-7DS*	21MY	*AX-110467729*	*AX-89378255*	3.24	6.91	−0.50	4.92	11.39	−5.85
	22GY			3.70	7.32	−0.50	4.19	8.26	−6.22

a LOD, logarithm of odds score.

b Add, additive effect of resistance allele.

c PVE, percentages of the phenotypic variance explained by individual QTL.

QTLs, quantitative trait loci; RILs, recombinant inbred lines.

### Identification of QTL resistance

**Figure 4:**
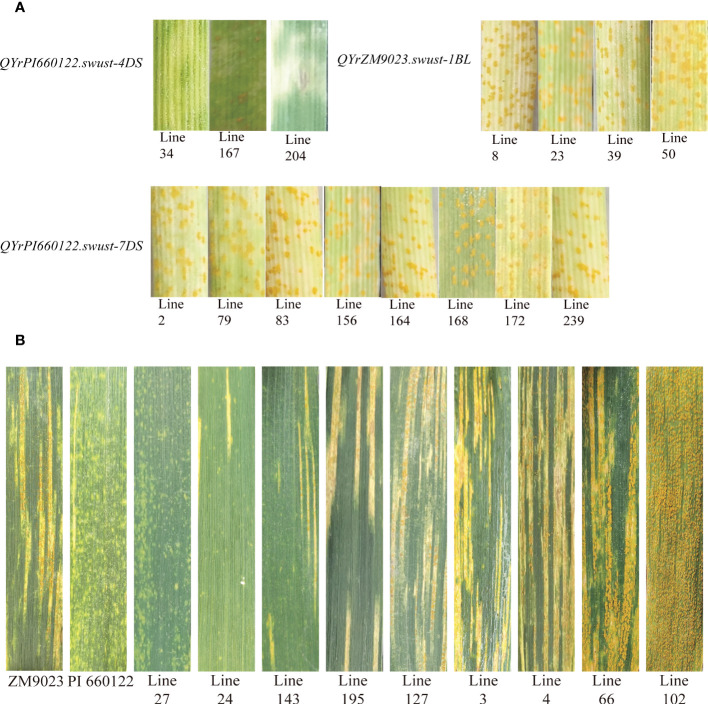
Stripe rust responses of lines containing only one QTL tested with Chinese race CYR32 of *Puccinia striiformis* f sp. *tritici* at the seedling stage **(A)** and on flag leaves of the resistant parent PI 660122, susceptible parent Zhengmai 9023 (ZM9023), and some selected recombinant inbred lines (RILs) **(B)**. Line 27 containing *QYrPI660122.swust-2BL*, *QYrPI660122.swust-4BS*, *QYrPI660122.swust-4BL*, *QYrPI660122.swust-4DS*, *QYrPI660122.swust-4DL*, *QYrZM9023.swust-6AS*, *QYrZM9023.swust-6DS*, and *QYrPI660122.swust-7DS*; Line 24 containing *QYrZM9023.swust-1BL*, *QYrPI660122.swust-2BL*, *QYrPI660122.swust-4BS*, *QYrPI660122.swust-4BL*, *QYrPI660122.swust-4DL*, and *QYrPI660122.swust-7DS*; Line 143 containing *QYrPI660122.swust-2BL*, *QYrPI660122.swust-4BS*, *QYrPI660122.swust-4BL*, *QYrPI660122.swust-4DL*, and *QYrPI660122.swust-7DS*; Line 195 containing *QYrPI660122.swust-4BS*, *QYrPI660122.swust-4BL*, *QYrPI660122.swust-4DL*, *QYrZM9023.swust-6AS*, and *QYrZM9023.swust-6DS*; Line 24 containing *QYrPI660122.swust-2BL* and *QYrPI660122.swust-4DS*; Line 3 containing *QYrZM9023.swust-1BL*, *QYrPI660122.swust-4BS*, *QYrPI660122.swust-4BL*, and *QYrPI660122.swust-4DL*; Line 4 containing *QYrPI660122.swust-2BL*, *QyrPI660122.swust-4BS*, *QyrPI660122.swust-4BL*, and *QyrPI660122.swust-4DL*; Line 66 and Line 102 without any QTLs. QTL, quantitative trait locus.

Fifteen lines containing only one QTL were selected and tested for seedling reaction in the greenhouse using three Chinese *Pst* races (CYR31, CYR32, and CYR34). Among them, four lines contained *QYrZM9023.swust-1BL*, three lines contained *QyrPI660122.swust-4DS*, and eight lines contained *QYrPI660122.swust-7DS*. The lines containing *QYrZM9023.swust-1BL* or *QYrPI660122.swust-7DS* were susceptible (IT of 7–9) to all three races but showed moderate resistance at the adult-plant stage in the fields, indicating that these QTLs confer APR. In contrast, the lines containing *QYrPI660122.swust-4DS* were resistant (IT of 1–3) in the seedling tests, indicating that this QTL confers ASR (**Figure 4**, **Table 5**). The resistance types of *QYrPI660122.swust-2BL*, *QYrPI660122.swust-4BS*, *QYrPI660122.swust-4BL*, *QYrPI660122.swust-4DL*, *QYrZM9023.swust-6AS*, and *QYrZM9023.swust-6DS* were uncertain, as there were no single-QTL lines available from the RIL population.

**Table 5:** Numbers of recombinant inbred lines from the Zhengmai 9023 × PI 660122 cross having only one stripe rust resistance and their infection types (ITs) at the seedling stage and mean IT and disease severity (DS) at the adult-plant stage in the fields of 2021 (21) and 2022 (22) at Mianyang (MY) and/or Guangyuan (GY).

QTL	No. of lines	Seedling ITs	Mean IT			Mean DS (%)		
			21MY	22MY	22GY	21MY	22MY	22GY
*QYrZM9023.swust-1BL*	4	8–9	5.6	7.1	5.8	39.5	36.2	40.0
*QYrPI660122.swust-4DS*	3	1–3	3.2	5.4	6.5	12.7	30.3	55.0
*QYrPI660122.swust-7DS*	8	7–9	6.4	7.3	5.9	26.6	40.2	42.5

QTL, quantitative trait locus.

### QTL combinations

**Figure 5:**
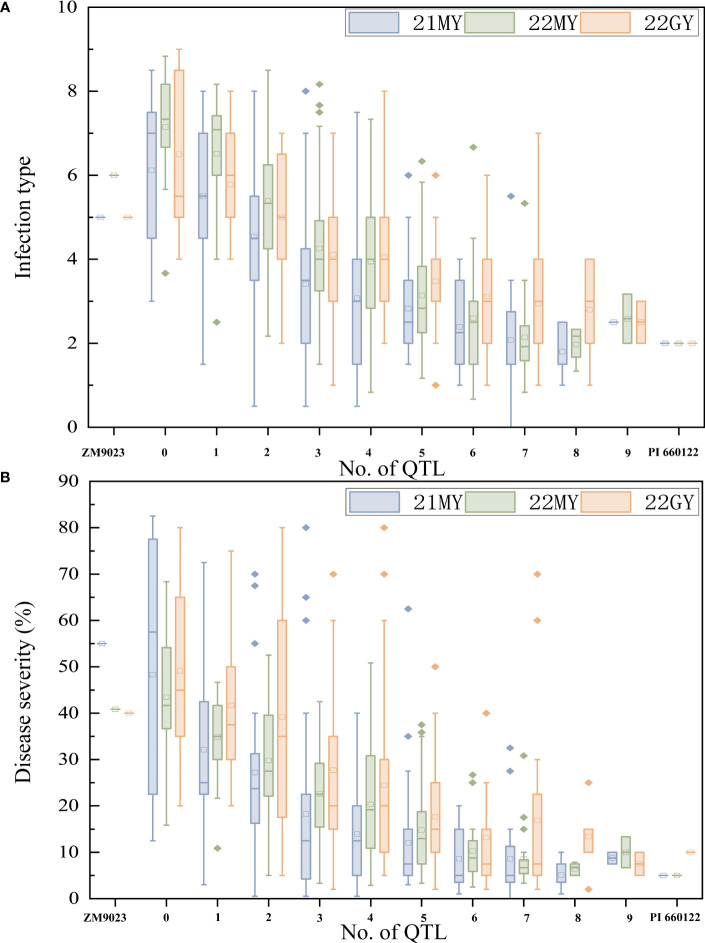
Effects of individual QTL and their combinations on stripe rust scores illustrated by mean infection type (IT) **(A)** and disease severity (DS) **(B)** scores of recombinant inbred lines from Zhengmai 9023 × PI 660122 (ZM9023 in three environments, 2021 Mianyang (21MY), 2022 Mianyang (22MY), and 2022 Guangyuan (22GY). Box plots indicate the infection type (IT) and disease severity (DS) associated with the identified QTL and their combination.

To determine the effects of the QTL in various combinations for *Pst* resistance, the 239 RILs were grouped into different genotypic groups based on the presence of markers closely associated with the nine QTL. These genotypes were further sorted into 10 groups based on the number of potential QTLs. Clearly, RILs carrying any number of QTL had lower mean DS than those without any of the QTL. Lines without any QTL had a mean IT of 6.6 and a mean DS of 46.91%. In comparison, when 0, 1, 2, 3, 4, 5, 6, and more than 6 QTLs were combined, the lines with one QTL had mean IT of 5.9 and mean DS of 36.04%, those with two QTLs had mean IT of 5.0 and mean DS of 31.81%, those with three QTLs had mean IT of 4.0 and mean DS of 23.25%, those with four QTLs had mean IT of 3.7 and mean DS of 19.52%, those with five QTLs had mean IT of 3.1 and mean DS of 14.76%, those with six QTLs had mean IT of 2.7 and mean DS of 10.45%, and those with seven or more QTL had mean IT of 2.4 and mean DS of 10.58%, close to the resistance level of PI 660122 (**Figure 5**).

### Selection of breeding lines

Various agronomic traits, including PH, PTN, SL, KPS, and TGW, of the parents and the 239 RILs were assessed in 2021 and 2022 in Mianyang and 2022 in Guangyuan. The mean PH values of PI 660122 and ZM9023 were 90.3 cm and 79.3 cm, respectively, and the RILs were mainly distributed in the range of 81–110 cm. The mean PTN values of PI 660122 and ZM9023 were 5 and 4, respectively, and the mean PTN values of RILs were between 4 and 10. The mean SL values of PI 660122 and ZM9023 were 9.8 cm and 8.4 cm, respectively, and the mean SL values of RILs were between 7.3 cm and 11.3 cm. The mean KPS values of PI 660122 and ZM9023 were 48 and 44, respectively, and the mean KPS values of RILs were between 33 and 58. The mean TGW values of PI 660122 and ZM9023 were 48 g and 44 g, respectively, and the mean TGW values of RILs were between 30.6 g and 58.4 g.

In order to select RILs with desirable agronomic traits, the following criteria were used: PH between 80 cm and 100 cm, PTN 5 or more, SL greater than 9 cm, KPS not less than 45, and TGW over 42 g, with stripe rust IT of 1–3 and DS < 20%. Based on these criteria, 26 lines were selected. The QTLs detected by their highly associated SNP markers in the selected lines are listed in **Table 6**. These lines had at least two QTLs, and three lines (F_6_-61, F_6_-78, and F_6_-86) had as many as seven QTLs. According to **Table 7**, the DS is negatively correlated with SL, PTN, KPS, and TGW, indicating that with the increase of DS, the SL, PTN, KPS, and TGW will decrease. The correlation coefficients between DS and PH, and PTN were 0.13 and 0.15, respectively, and the correlation was significant (*p* < 0.05). The correlation coefficients between DS and SL, KPS, and TGW were 0.05, 0.08, and 0.02, respectively, and the correlation was not significant (*p* > 0.05).

**Table 6:** Mean stripe rust response, agronomic traits, and presence (+) and absence (−) of resistant QTLs detected with SNP markers in Zhengmai (ZM) 9023, PI 661022, and selected recombinant inbred lines^a^.

Parent/line	Stripe rust		Agronomic trait					QTL								
	IT	DS (%)	PH	SL	PTN	KPS	TGW	1BL	2BL	4BS	4BL	4DS	4DL	6AS	6DS	7DS
ZM9023	5	45	79.3	8.4	4	44	40.9	+	−	−	−	−	−	+	+	−
PI 660122	2	7	90.3	9.8	5	49	45.1	−	+	+	+	+	+	−	−	+
F_6_-5	2	9	95.0	9.5	5	49	46.4	+	−	+	+	+	+	−	−	−
F_6_-13	1	3	93	9.7	5	50	52.1	−	+	+	+	+	+	−	−	−
F_6_-18	3	13	83.0	9.4	6	48	53.7	+	−	−	−	−	−	+	+	−
F_6_-22	2	5	97.0	9.1	7	46	47.5	+	+	+	+	−	+	−	−	+
F_6_-27	1	3	93.7	9.4	8	52	50.7	−	+	+	+	+	+	+	+	+
F_6_-32	3	9	99.3	10.4	6	52	56.8	+	+	−	−	−	−	−	−	+
F_6_-48	3	11	95.7	9.1	6	51	46.9	+	−	−	−	+	−	−	−	+
F_6_-54	2	7	84.3	9.1	6	49	45.0	−	−	+	+	−	+	−	−	−
F_6_-56	2	5	83.7	9.0	6	45	53.5	−	−	−	−	+	−	+	+	+
F_6_-61	3	10	86.7	10.2	7	52	46.3	+	−	+	+	+	+	+	+	−
F_6_-78	2	6	96.3	9.5	6	49	44.2	−	+	+	+	+	+	+	+	−
F_6_-86	3	14	90	9.6	7	50	47.9	+	+	+	+	−	+	+	+	−
F_6_-115	3	12	98	9.7	5	48	52.6	+	+	−	−	+	+	−	−	+
F_6_-124	2	6	90.7	9.4	8	57	53.2	+	+	−	−	−	−	+	+	−
F_6_-150	3	13	98	9.6	6	47	51.9	−	+	−	−	+	−	+	+	−
F_6_-161	3	15	93	9.4	7	54	50.1	−	−	−	−	−	−	−	+	+
F_6_-162	3	11	94.7	9.1	7	49	49.5	−	+	−	−	+	−	−	−	−
F_6_-170	3	11	93	9.6	7	50	52.0	−	+	−	−	−	−	+	+	+
F_6_-178	3	11	89.5	9.5	7	51	48.3	+	−	+	−	−	+	+	+	−
F_6_-186	2	8	96	9.9	5	45	46.0	−	−	−	−	+	−	−	−	+
F_6_-187	2	4	80	9.4	5	49	52.4	+	−	+	+	+	+	−	−	−
F_6_-207	2	3	82	9.7	6	52	44.0	−	−	+	+	+	+	+	+	+
F_6_-211	2	3	82.0	9.7	6	52	49.6	−	−	+	−	+	+	−	−	−
F_6_-228	3	10	92.7	9.5	6	48	49.3	−	−	−	−	+	−	+	+	+
F_6_-231	3	14	93.7	9.3	5	56	45.6	+	−	−	−	−	−	+	+	+
F_6_-235	3	13	97	9.5	7	48	49.9	+	+	−	−	+	−	+	+	−

a IT, infection type; DS, disease severity; PH, plant height; PTN, productive tiller number; SL, spike length; KPS, kernels per spike; TGW, thousand-grain weight.

QTLs, quantitative trait loci; SNP, single-nucleotide polymorphism.

**Table 7:** Correlation coefficients (r) of important traits of the recombinant inbred lines Zhengmai 9023 × PI 660122.

Trait	IT	DS (%)	PH	SL	PTN	KPS	TGW
IT	NA^a^						
DS (%)	0.95***^b^	NA					
PH	0.16*	0.13*	NA				
SL	−0.01ns	−0.05	0.10ns	NA			
PTN	−0.12ns	−0.15*	−0.02ns	0.07ns	NA		
KPS	−0.03ns	−0.08ns	0.19**	0.45***	0.12ns	NA	
TGW	0.01ns	−0.02ns	0.06ns	0.06ns	0.02ns	−0.02ns	NA

a NA, not applicable.

b “***” denotes the r value is significant at <0.001, “**” denotes the r value is significant at <0.01, “*” denotes the r value is significant at <0.05, and “ns” denotes the r value is not significant at p > 0.05.

## Discussion

Developing durable resistance to stripe rust has been a top priority in wheat breeding over the past decade. Wheat line PI 660122 has exhibited high levels of stripe rust resistance for over a decade (Wang et al., 2012). In the present study, a RIL population containing 239 lines developed from a cross of PI 660122 with a Chinese elite cultivar, ZM9023, was phenotyped for stripe rust response in multiple environments and genotyped with the 15K wheat SNP array. We detected a total of nine QTLs, of which six (*QYrPI660122.swust-2BL*, *QyrPI660122.swust-4BS*, *QyrPI660122.swust-4BL*, *QyrPI660122.swust-4DS*, *QyrPI660122.swust-4DL*, and *QyrPI660122.swust-7DS*) came from PI 660122 and three (*QYrZM9023.swust-1BL*, *QYrZM9023.swust-6AS*, and *QYrZM9023.swust-6DS*) came from ZM9023.

*QYrZM9023.swust-1BL*, an APR QTL, was from Zhengmai 9023. This QTL was flanked by SNP markers *AX-89763895* and *AX-109273019*, corresponding to the region from 670,429,611 bp to 681,685,826 bp of chromosome 1BL in the Chinese Spring (CS) genome (IWGSC RefSeq v1.0). Three permanently named stripe rust resistance genes, *Yr21* (Chen and Line, 1995), *Yr26* (Ma et al., 2001), and *Yr29* (William et al., 2006), were mapped to 1BL. Among them, *Yr21* and *Yr26* confer ASR, while *Yr29* is an APR gene linked with SSR markers *Xwmc44* and *Xwmc367* and located at the distal end of chromosome 1BL. The physical position of *Xwmc367* is in the range of 678,736,681–678,736,834 bp, which is within the genome range of *QYrZM9023.swust-1BL*. According to a previous study, ZM9023 has *Lr27*/*Yr30*/*Sr2* and *Lr46*/*Yr29*/*Pm39*/*Sr58* (Li et al., 2020). As both *Yr29* and *QYrZM9023.swust-1BL* confer APR, *QYrZM9023.swust-1BL* should be *Yr29*. *Yr29* has been reported and deployed widely in wheat varieties around the world (William et al., 2006; Melichar et al., 2008; Lan et al., 2014; Hou et al., 2015; Maccaferri et al., 2015; Muleta et al., 2017; Wang and Chen, 2017; Cobo et al., 2018; Godoy et al., 2018; Liu et al., 2019; Rosa et al., 2019; Liu et al., 2020).

*QYrPI660122.swust-2BL* was derived from PI 660122, and it was flanked by SNP markers *AX-109849173* and *AX-109349804*, corresponding to the region from 777,831,275 bp to 779,847,527 bp of chromosome 2BL in CS (IWGSC RefSeq v1.0). Seven permanently named stripe rust resistance genes, including *Yr5* and *Yr7* (Macer, 1963; Marchal et al., 2018), *Yr43* (Cheng and Chen, 2010), *Yr44* (Sui et al., 2009), *Yr53* (Xu et al., 2013), *Yr72* (McIntosh et al., 2016), and *YrSP* (Feng et al., 2015), were mapped to 2BL. *Yr5*/*Yr7*/*YrSP* (Marchal et al., 2018) had been cloned, and it encoded nucleotide-binding (NB) and leucine-rich repeat (LRR) proteins. The physical map position of *Yr5*/*Yr7*/*YrSP* was 685.265–685.27 Mb. *Yr43*, an ASR gene, was flanked by *Xwgp110* and *Xwgp103*, but its physical map position is unknown. *Yr44*, an ASR gene, was derived from spring wheat cultivar Zak and flanked by *XpWB5/N1R1* and *Xwgp100*, but its physical map position is unknown. *Yr53*, an ASR gene, was derived from PI 480148 and flanked by *Xwmc441* and *XLRRrev*/*NLRRrev350*. The physical map position of *Xwmc441* was 598,064,318–598,064,477 bp. *Yr72*, an ASR gene, was derived from AUS27507 and flanked by *Xsun481* and *IWB12294*. The physical map position of *IWB12294* was 767,171,587–767,171,587 bp. In addition, several major QTLs have been mapped to the long arm of chromosome 2B. *QYr.hbaas-2BL* was located at 453.3 Mb (Jia et al., 2020). *Yr.niab-2B.1* was located at 683.05–750.12 Mb (Bouvet et al., 2022). *QYrpd.Swust-2BL.1*, *QYrpd.Swust-2BL.2*, *QYrpd.Swust-2BL.3*, and *QYrpd.Swust-2BL.4* were located at 773.79–775.17 Mb, 753.37–777.52 Mb, 793.15–798.00 Mb, and 782.53–784.55 Mb, respectively (Zhou et al., 2022b). *Qyr.gaas.2B.1* was located at 698.22–705.68 Mb (Cheng et al., 2022). *YrQz* was located at 557.37–630.40 Mb (Deng et al., 2004). *QYr.nafu-2BL* was located at 553.73–615.79 Mb (Zhou et al., 2015a; Hu et al., 2020). *QYrww.wgp.2B-4* was located at 524 Mb (Mu et al., 2020). *Yrdr.wgp-2BL* was located at 709.84 Mb (Hou et al., 2015). *QTL 2BL* was located at 779.11–783.89 Mb (Somers et al., 2004). *QYr.inra-2BL* was located at 615.79–621.47 Mb (Mallard et al., 2005). *QYraq.cau-2BL* was located at 670.60–739.40 Mb (Ramburan et al., 2004). *QYr.caas-2BL* was located at 693.74–733.16 Mb (Ren et al., 2012b). *Yrns.orz-2BL* was located at 685.74 Mb (Vazquez et al., 2015). *YrV23* is closely linked to *Xwmc356* at position 796,684,893–796,685,357 bp (Wang et al., 2006). Based on the chromosomal positions, *QYrPI660122.swust-2BL* is likely different from *Yr5*/*Yr7*/*YrSP* and *Yr53*, but its relationships with other genes or QTL on 2BL need to be further studied.

*QYrPI660122.swust-4BS* was derived from PI 660122, and it was flanked by SNP markers *AX-108767762* and *AX-109309162*, corresponding to the region from 32,961,964 bp to 36,395,734 bp of the CS (IWGSC RefSeq v1.0) chromosome 4BS. *QYr.caas-4BS* was located between markers *Xwmc652* and *Xgpw4388* (Wang et al., 2020). The physical map position of *QYr.caas-4BS* was 38.6–47.6 Mb. *QYrcl.sicau-4B* was located at the end of chromosome 4BS (Yao et al., 2020), which is different from the physical map position of *QYrPI660122.swust-4BS*. *QYrPI660122.swust-4BS* in PI 660122 is likely different from the previously mapped stripe rust resistance genes on chromosome 4BS.

*QYrPI660122.swust-4BL* was derived from PI 660122 and was flanked by SNP markers *AX-109495166* and *AX-108935256*, corresponding to the position from 396,263,280 bp to 445,689,397 bp of the CS chromosome 4BL. Three permanently named stripe rust resistance genes, *Yr50* (Liu et al., 2013), *Yr62* (Lu et al., 2014), and *Yr68* (McIntosh et al., 2016), were mapped to 4BL. *Yr50*, an ASR gene, was reported to be associated with *Xbarc1096* and *Xwmc47*. The map position of *Yr50* is 105.1 Mb–644.9 Mb. *Yr62*, a HTAP gene, was reported to be associated with *Xgwm192* and *Xgwm251*. The physical map position of *Yr62* was 482.8–568.6 Mb. *Yr68*, an APR gene, was reported to be associated with *IWB74301* and *IWB28394*, and its physical map position was 575.04–600.66 Mb. In addition, several major QTLs were located on chromosome 4BL. *QYrhm.nwafu-4B*, *Qyr.hbaas-4BL.1*, and *QYr.hbaas-4BL.2* overlapped *Yr62* (Yuan et al., 2018; Jia et al., 2020). *QYrhm.nwafu-4BL* was derived from Humai 15 and flanked by *AX-111150955* and *Xgwm251*, which was mapped to 523.4–568.6 Mb (Yuan et al., 2018). *QYr.hbaas-4BL.1* was linked with *IWB73717*, and its physical map position was 531.3 Mb (Jia et al., 2020). *QYr.hbaas-4BL.2* was linked with *IWB63337* at the physical map position of 558.1 Mb. *QYr.hbaas-4BL.3* was linked with *IWB32927* at the physical map position of 579.4 Mb. *QYr.sun-4B* was derived from the Australian wheat cultivar Janz and exhibited minor variation (9.4%–16.8%) (Zwart et al., 2010). It was flanked by *wPt-8543* and *Xwmc238*. The physical map position of *Xwmc238* was 236,742,906–236,743,133 bp. *QPst.jic-4B* (Melichar et al., 2008) was derived from the UK winter wheat cultivar Guardian and mapped to the region between *Xwmc652* and *Xwmc692* with a PVE of 12%. *QYr.crc-4BL* was flanked by markers *BS00048794_51* and *RAC875_rep_c72961_977*, and the physical map position of *QYr.crc-4BL* was 601.93–617.00 Mb (Rosa et al., 2019). *YrBm*, an APR QTL, was derived from Chinese winter wheat landrace Baimangmai (Hu et al., 2022). It was flanked by markers *Xgpw7272* and *Xwmc652*. The physical map position of *YrBm* was 611.1–621.1 Mb. *QYrPI660122.swust-4BL* overlapped with *Yr50*, but further studies are needed to confirm the relationship between *QYrPI660122.swust-4BL* and *Yr50* and determine the relationships with other QTLs on chromosome 4BL.

*QYrPI660122.swust-4DS*, an ASR QTL, was derived from PI 660122, and it was flanked by SNP markers *AX-110046962* and *AX-111093894* and corresponds to the region from 1,702,954 bp to 9,555,772 bp of the CS 4DS chromosome. *Yr28* has been mapped to the short arm of chromosome 4D (Singh et al., 2000). *Yr28* is a major ASR gene conferring stripe rust resistance from *Ae. tauschii* and located between SSR markers *Xbcd265* and *Xmwg634*. *Yr28* has been cloned and characterized, which encoded a typical nucleotide oligomerization domain-like receptor (NLR) (Zhang et al., 2019). The gene was further mapped between *Xsdauw92* and *Xsdauw96*, approximately 0.13-cM interval, and its physical map position of *Yr28* was 1.820–1.826 Mb. Based on the physical map position, the resistance type, and the Mexican wheat genotype PI 610755 that has *Ae. tauschii* in the pedigree as stripe rust resistance donor of PI 660122 (Wang et al., 2012), *QYrPI660122.swust-4DS* is highly likely *Yr28*.

*QYrPI660122.swust-4DL* was derived from PI 660122 and was flanked by SNP markers *AX-94560848* and *AX-111557122* corresponding to 288,430,275 bp to 310,458,135 bp of the CS chromosome 4DL. So far, only one permanently named *Yr* gene, *Yr46* (Herrera-Foessel et al., 2011), has been reported on chromosome 4DL. *Yr46* is an APR gene from wheat cultivar RL6007 and flanked by SSR markers *Xgwm165* and *Xgwm192*, and its physical map position is approximately 417.2 Mb. Two QTLs, *QYr.ucw-4DL* (Cobo et al., 2018) and *QYr.hbaas-4DL* (Jia et al., 2020), have been also reported on chromosome 4DL. *QYr.ucw-4DL* was linked with the *IWA2395*, and its physical map position was 497.65 Mb (Cobo et al., 2018). *QYr.hbaas-4DL* is linked to SNP marker *IWB44356* (Jia et al., 2020), with the physical map position of approximately 477.9 Mb. Based on the different physical map positions of *QYrPI660122.swust-4DL* from those of *Yr46*, *QYr.ucw-4DL*, and *QYr.hbaas-4DL*, *QYrPI660122.swust-4DL* is likely a new QTL for stripe rust resistance.

*QYrZM9023.swust-6AS* is derived from Zhengmai 9023 and flanked by SNP markers *AX-95124889* and *AX-110995858* corresponding to the 27,748,586–71,705,701-bp region of the CS chromosome 6AS. Numerous genes or QTLs for stripe rust resistance have been mapped to 6AS. *Yr38* was mapped to 6AS (Marais et al., 2006), but its physical map position is unknown. *Yr81* is flanked by *KASP_3077* and *Xgwm459* (Gessese et al., 2019), and the physical map position of *Xgwm459* is within the 6,805,513–6,805,994-bp region. *YrP10090* is flanked by *AX-94460938* and *AX-110585473* (Liu et al., 2021), and its physical map position is within 107.1–446.5 Mb. *Qyr.gaas.6A* was flanked by *AX-109558600* and  *AX-109542604* (Cheng et al., 2022), and its physical map position is within 609.11–609.89 Mb. *QYr-6A_Saar* derived from the CIMMYT variety Saar (Lillemo et al., 2008) is flanked by *XwPt-7063* and *Xbarc3*, and its physical map position is within 62.92–85.28 Mb. *QYr.uaf-6A.1* and *QYr.uaf-6A.4* were mapped with *IWA8028* and *IWB29623*, respectively (Habib et al., 2020). The physical map position of *QYr.uaf-6A.1* is at 105.02 Mb, and that of *QYr.uaf-6A.4* is at 18.71 Mb. *QYr.uga-6AS* is flanked by *wPt-671561* and *wPt-7840* (Hao et al., 2011), and its physical map position is within 24.09–85.28 Mb. *QYrex.wgp-6AS* is flanked by markers *Xgwm334* and *Xwgp56* (Lin and Chen, 2008a), which indicate the QTL physical map position within 9.25–61.02 Mb. *Yrq3* is flanked by SSR markers *Xgwm334* and *Xgwm169* (Cao et al., 2012), and its physical map position was found to be within 9.25–595.38 Mb. *QYrcl.sicau-6A.1* was detected by two adjacent DArT-seq markers (*3936688* and *1266956*) on chromosome 6A between 5.14 and 5.83 Mb (Yao et al., 2020). *QYr.sicau-6A* is flanked by SNP markers *AX-94548199* and *AX-111101235* (Ma et al., 2019), and its physical map position of *QYr.sicau-6A* is within 90.32–97.52 Mb. *QYrswp-6A* is linked with *IWA272* (Liu et al., 2019b), and its physical map position is 3.85 Mb. *QYrZM9023.swust-6AS* overlapped with *QYr-6A_Saar*, *Yr.uga-6AS*, and *QYrex.wgp-6AS*, but further studies are needed to determine if they are the same or different.

*QYrZM9023.swust-6DS* was also derived from Zhengmai 9023 and flanked by SNP markers *AX-11475193* and *AX-109317417* corresponding to the 35,630,857–44,498,347-bp region of the CS chromosome 6DS. Few genes or QTL for stripe rust resistance have been reported on 6DS. *Yr77* is an APR gene flanked by *Xbrac54* and *Xcfd188*, and the physical map position of *Xcfd188* is within the 238,118,148–238,118,395-bp region (R. McIntosh, personal communication). *QYr.ucw-6D* is linked with *IWA167* (Maccaferri et al., 2015), which is at the physical map position of 73.2 Mb. *QYr.ufs-6D* is flanked by *Xgwm325* and *Xbarc175* (Agenbag et al., 2012), and its physical map position is within the 79.96–411.88-Mb region. *QYR7* is flanked by *Xbcd1510* and *XksuD27* (Boukhatem et al., 2002), and its physical map position is approximately 12 Mb. Based on the physical positions, *QYrZM9023.swust-6DS* is likely different from these genes or QTLs for stripe rust resistance genes previously mapped on chromosome 6DS.

*QYrPI660122.swust-7DS*, an APR QTL derived from PI 660122, is flanked by SNP markers *AX-110467729* and *AX-89378255* corresponding to the 43,386,933–47,379,368-bp region of the CS 7DS chromosome. Only the permanently named stripe rust resistance gene *Yr18* has been mapped to chromosome 7DS. *Yr18* was mapped to 7DS in a number of different wheat cultivars, such as Jupateco 73R and Opata 85 (Singh, 1992; Singh et al., 2000), Australian cultivar Cook (Bariana et al., 2001), and Fukuho-komugi (Suenaga et al., 2003). *Yr18* is an APR gene flanked by *Xgwm1220* and *Xgwm29* and encodes a putative ATP-binding cassette transporter (Krattinger et al., 2009; Lagudah et al., 2009). The physical map position of *Yr18* is from 47.412 Mb to 47.424 Mb, similar to *QYrPI660122.swust-7DS*. *Yr18* is an important slow rusting gene and can confer high levels of resistance when combined with other minor genes (Singh and Rajaram, 1993; Navabi et al., 2004). Cultivars with *Yr18* have been widely used in the International Maize and Wheat Improvement Center (CIMMYT) wheat breeding program (Singh et al., 2005). PI 660122 was developed from a cross of AvS with Mexican wheat genotype PI 610755 (Wang et al., 2012), PI 610755 has Opata 85 in its pedigree (Altar 84/*Ae. tauschii* (191)//Opata M85) (https://npgsweb.ars-grin.gov/gringlobal/accessiondetail?id=1580210), *Yr18* was mapped in a RIL population for mapping *Yr28* (Singh et al., 2000) as discussed above, and *QYrPI660122.swust-7DS* is most likely *Yr18*.

## Conclusions

In the present study, we mapped nine QTLs conferring different types and levels of resistance to stripe rust. Among these QTLs, *QYrZM9023.swust-1BL* was identified as *Yr29*, *QYrPI660122.swust-4DS* as *Yr28*, and *QYrPI660122.swust-7DS* as *Yr18*, while *QYrPI660122.swust-4BS*, *QYrPI660122.swust-4BL*, and *QYrZM9023.swust-6DS* should be new. We demonstrated that combinations of different QTLs increased the levels of resistance. Furthermore, we selected lines from the RIL population with high adequate resistance to stripe rust combined with desirable agronomic traits, and these lines can be used in further evaluation for releasing commercial cultivars. The resistant lines and molecular markers for resistance QTL should be useful in developing wheat cultivars with high levels and durable resistance to stripe rust.

## Data availability statement

The original contributions presented in the study are included in the article/supplementary material, further inquiries can be directed to the corresponding author/s.

## Author contributions

QY and GJ detected the QTLs, analyzed the data, and prepared the first draft of the manuscript. QY, WT, RT, XQL, JF, XCZ and BS contributed to the collection of samples and phenotype data. XLZ contributed to the crosses and revised the manuscript. YR, XL, KH, and SY contributed to target line selection and population assessment. MW and XC provided seeds of the stripe rust-resistant parent. XLZ and XC conceived the project and generated the final version of the manuscript. All authors provided suggestions during the revision of the manuscript. All authors contributed to the article and approved the submitted version.
